# Tris(diisopropyl­ammonium) hydrogensulfate sulfate

**DOI:** 10.1107/S160053680802237X

**Published:** 2008-07-23

**Authors:** Gholamhossein Sh. Mohammadnezhad, Mostafa M. Amini, Hamid Reza Khavasi, Seik Weng Ng

**Affiliations:** a Department of Chemistry, Shahid Beheshti University, Tehran, Iran; b Department of Chemistry, University of Malaya, 50603 Kuala Lumpur, Malaysia

## Abstract

The cations and anions of the title salt, 3C_6_H_16_N^+^·HSO_4_^−^·SO_4_^2−^, are linked by N—H⋯O and O—H⋯O hydrogen bonds into a three-dimensional network. The hydrogensulfate ion, with a single S—O(H) bond of 1.563 (2) Å, forms a short O—H⋯O hydrogen bond [O⋯O = 2.609 (2) Å] to the sulfate ion. The hydrogensulfate ion accepts two hydrogen bonds from two cations, whereas the sulfate ion, as an acceptor, binds to four cations. The sulfate ion is disordered approximately equally over two sites related by rotation around one of the O—S bonds.

## Related literature

For the crystal structures of other hydrogensulfate–sulfate salts, see: Anderson *et al.* (2006 ▶); Banerjee & Murugavel (2004 ▶); Kang *et al.* (2005 ▶); Novozhilova *et al.* (1987 ▶); Sridhar *et al.* (2001 ▶); Warden *et al.* (2004 ▶). For the synthesis of ammonium sulfates, see: Jordanovska *et al.* (2000 ▶).
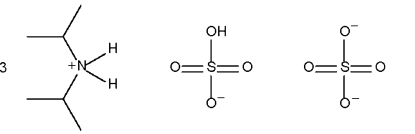


## Experimental

### 

#### Crystal data

3C_6_H_16_N^+^·HSO_4_^−^·SO_4_^2−^
*M* *_r_* = 499.72
Monoclinic, 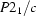

*a* = 8.6178 (6) Å
*b* = 16.741 (1) Å
*c* = 19.819 (1) Å
β = 101.973 (5)°
*V* = 2797.2 (3) Å^3^
*Z* = 4
Mo *K*α radiation
μ = 0.23 mm^−1^
*T* = 295 (2) K
0.40 × 0.30 × 0.25 mm

#### Data collection

Stoe IPDSII imaging plate diffractometer
Absorption correction: analytical (*X-SHAPE*; Stoe & Cie, 2003 ▶) *T*_min_ = 0.91, *T*_max_ = 0.94
16154 measured reflections
6311 independent reflections
4905 reflections with *I* > 2σ(*I*)
*R*_int_ = 0.035

#### Refinement

*R*[*F* ^2^ > 2σ(*F* ^2^)] = 0.049
*wR*(*F* ^2^) = 0.122
*S* = 1.06
6311 reflections
336 parameters
94 restraints
H atoms treated by a mixture of independent and constrained refinement
Δρ_max_ = 0.26 e Å^−3^
Δρ_min_ = −0.23 e Å^−3^

### 

Data collection: *X-RED* (Stoe & Cie, 2001 ▶); cell refinement: *X-AREA* (Stoe & Cie, 2005 ▶); data reduction: *X-AREA*; program(s) used to solve structure: *SHELXS97* (Sheldrick, 2008 ▶); program(s) used to refine structure: *SHELXL97* (Sheldrick, 2008 ▶); molecular graphics: *X-SEED* (Barbour, 2001 ▶); software used to prepare material for publication: *SHELXL97*.

## Supplementary Material

Crystal structure: contains datablocks global, I. DOI: 10.1107/S160053680802237X/gk2158sup1.cif

Structure factors: contains datablocks I. DOI: 10.1107/S160053680802237X/gk2158Isup2.hkl

Additional supplementary materials:  crystallographic information; 3D view; checkCIF report

## Figures and Tables

**Table 1:** Hydrogen-bond geometry (Å, °)

*D*—H⋯*A*	*D*—H	H⋯*A*	*D*⋯*A*	*D*—H⋯*A*
O7—H7⋯O4	0.86 (1)	1.76 (1)	2.609 (2)	174 (4)
N1—H1*N*1⋯O1	0.86 (1)	1.76 (1)	2.585 (5)	159 (3)
N1—H1*N*2⋯O6^i^	0.85 (1)	2.02 (1)	2.874 (2)	176 (3)
N2—H2*N*2⋯O4	0.85 (1)	2.10 (1)	2.929 (2)	166 (2)
N2—H2*N*1⋯O2^ii^	0.85 (1)	1.85 (1)	2.695 (6)	179 (3)
N2—H2*N*1⋯O2′^ii^	0.85 (1)	2.02 (2)	2.855 (8)	166 (3)
N3—H3*N*1⋯O3	0.86 (1)	1.89 (1)	2.738 (8)	174 (2)
N3—H3*N*1⋯O3′	0.86 (1)	1.88 (1)	2.711 (7)	162 (2)
N3—H3*N*2⋯O5^iii^	0.86 (1)	2.00 (1)	2.819 (2)	159 (2)

Symmetry codes: (i) 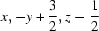; (ii) 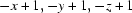; (iii) 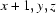.

**Fig. 1.**
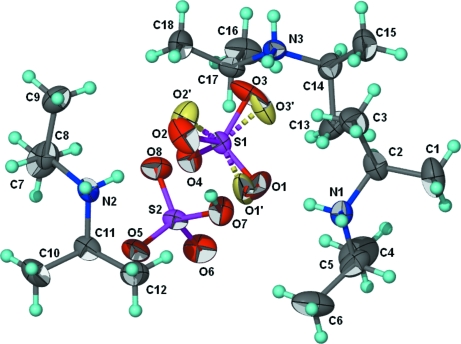
Molecular structure of [(C6H16N)3]3 [HSO4] [SO4]; displacement ellipsoids are drawn at the 50% probablity level and H atoms are drawn as spheres of arbitrary radii. Disorder in the sulfate ion is shown.

## supplementary crystallographic information

### Comment

Disubstituted ammonium sulfates are used in the synthesis of double salts with other metal sulfates (Jordanovska *et al.*, 2000). The reaction of diisopropylamine with sulfuric acid yielded the expected compound as a double sulfate with diisopropylammonium hydrogensulfate ( Fig. 1). A small number of such double salts are known (Anderson *et al.*, 2006; Banerjee & Murugavel, 2004; Kang *et al.*, 2005; Novozhilova *et al.*, 1987; Sridhar *et al.*, 2001; Warden *et al.*, 2004). In the title compound, the cations and anions are linked by N–H···O and O–H···O hydrogen bonds into three-dimensional network structure. The sulfate ion is disordered over two sites in an "umbrella" type of disorder (only three of the four oxygen atoms are disordered).

### Experimental

Following the method of Jordanovska *et al.* (2000), diisopropylamine (1 ml, 7.1 mmol) was dissolved in chloroform (10 ml) and concentrated sulfuric acid was added dropwise at 273 K until a white precipitate was formed. The precipitate was collected and recrystallized from water.

### Refinement

The sulfate ion is disordered over two positions related by rotation around the S1-O4 bond. For this ion, all S–O distances were restrained to be equal within 0.01 Å. Similar restraints were were imposed on O···O distances within this ion.

Carbon-bound hydrogen atoms were placed in calculated positions (C–H 0.96 – 0.98 Å), and were included in refinement in the riding model approximation, with *U*(H) set to 1.2-1.5 times *U*_eq_(C). Oxygen and nitrogen-bound hydrogen atoms were located in a difference Fourier map, and were refined with a distance restraint (O,N)–H= 0.85±0.01 Å; their temperature factors were freely refined.

### Crystal data

**Table:**

3C_6_H_16_N^+^·HSO_4_^–^·SO_4_^2–^	*F*_000_ = 1096
*M**_r_* = 499.72	*D*_x_ = 1.187 Mg m^−^^3^
Monoclinic, *P*2_1_/*c*	Mo *K*α radiation λ = 0.71073 Å
Hall symbol: -P 2ybc	Cell parameters from 4365 reflections
*a* = 8.6178 (6) Å	θ = 2.4–27.5º
*b* = 16.741 (1) Å	µ = 0.23 mm^−^^1^
*c* = 19.819 (1) Å	*T* = 295 (2) K
β = 101.973 (5)º	Block, colorless
*V* = 2797.2 (3) Å^3^	0.40 × 0.30 × 0.25 mm
*Z* = 4	

### Data collection

**Table:**

Stoe IPDS-II imaging plate diffractometer	6311 independent reflections
Radiation source: medium-focus sealed tube	4905 reflections with *I* > 2σ(*I*)
Monochromator: graphite	*R*_int_ = 0.035
*T* = 295(2) K	θ_max_ = 27.5º
Rotation method scans	θ_min_ = 2.4º
Absorption correction: analytical(X-SHAPE; Stoe & Cie, 2003)	*h* = −11→11
*T*_min_ = 0.91, *T*_max_ = 0.94	*k* = −21→21
16154 measured reflections	*l* = −25→15

### Refinement

**Table:**

Refinement on *F*^2^	Secondary atom site location: difference Fourier map
Least-squares matrix: full	Hydrogen site location: inferred from neighbouring sites
*R*[*F*^2^ > 2σ(*F*^2^)] = 0.049	H atoms treated by a mixture of independent and constrained refinement
*wR*(*F*^2^) = 0.122	*w* = 1/[σ^2^(*F*_o_^2^) + (0.054*P*)^2^ + 0.953*P*] where *P* = (*F*_o_^2^ + 2*F*_c_^2^)/3
*S* = 1.06	(Δ/σ)_max_ = 0.001
6311 reflections	Δρ_max_ = 0.26 e Å^−^^3^
336 parameters	Δρ_min_ = −0.23 e Å^−^^3^
94 restraints	Extinction correction: none
Primary atom site location: structure-invariant direct methods	

### Fractional atomic coordinates and isotropic or equivalent isotropic displacement parameters (Å^2^)

**Table:**

	*x*	*y*	*z*	*U*_iso_*/*U*_eq_	Occ. (<1)
S1	0.69023 (5)	0.61641 (3)	0.59691 (2)	0.03414 (13)	
S2	0.50949 (5)	0.60829 (3)	0.81272 (3)	0.03843 (14)	
O1	0.6113 (17)	0.6922 (6)	0.5791 (8)	0.093 (4)	0.49 (3)
O2	0.6440 (16)	0.5588 (7)	0.5417 (4)	0.068 (3)	0.49 (3)
O3	0.8606 (6)	0.6263 (9)	0.6126 (4)	0.072 (3)	0.49 (3)
O4	0.63401 (18)	0.58408 (10)	0.65737 (8)	0.0505 (4)	
O1'	0.5800 (8)	0.6754 (5)	0.5608 (5)	0.082 (3)	0.51 (3)
O2'	0.6958 (17)	0.5496 (4)	0.5505 (4)	0.065 (2)	0.51 (3)
O3'	0.8453 (7)	0.6519 (7)	0.6185 (4)	0.068 (2)	0.51 (3)
O5	0.36497 (17)	0.57133 (11)	0.77530 (9)	0.0522 (4)	
O6	0.4807 (2)	0.66251 (12)	0.86590 (9)	0.0640 (5)	
O7	0.5682 (2)	0.66486 (10)	0.76049 (10)	0.0562 (4)	
H7	0.584 (4)	0.640 (2)	0.7247 (12)	0.099 (12)*	
O8	0.63320 (19)	0.55114 (12)	0.83566 (10)	0.0625 (5)	
N1	0.6123 (2)	0.82382 (11)	0.51105 (9)	0.0410 (4)	
H1n1	0.604 (3)	0.7752 (8)	0.5238 (13)	0.046 (6)*	
H1n2	0.577 (3)	0.8265 (16)	0.4676 (6)	0.057 (7)*	
N2	0.4034 (2)	0.46240 (11)	0.59581 (9)	0.0378 (4)	
H2n1	0.390 (3)	0.4557 (16)	0.5525 (6)	0.059 (8)*	
H2n2	0.480 (2)	0.4947 (12)	0.6089 (13)	0.049 (7)*	
N3	1.0587 (2)	0.63815 (11)	0.73947 (9)	0.0379 (4)	
H3n1	1.003 (2)	0.6345 (14)	0.6985 (7)	0.047 (7)*	
H3n2	1.1482 (17)	0.6160 (13)	0.7392 (13)	0.044 (6)*	
C1	0.8175 (4)	0.92758 (18)	0.50748 (18)	0.0813 (9)	
H1A	0.7674	0.9628	0.5347	0.122*	
H1B	0.7749	0.9369	0.4594	0.122*	
H1C	0.9297	0.9374	0.5172	0.122*	
C2	0.7866 (3)	0.84177 (15)	0.52506 (12)	0.0518 (6)	
H2	0.8301	0.8333	0.5743	0.062*	
C3	0.8635 (3)	0.78284 (19)	0.48440 (18)	0.0711 (8)	
H3A	0.8421	0.7294	0.4975	0.107*	
H3B	0.9761	0.7916	0.4938	0.107*	
H3C	0.8213	0.7900	0.4360	0.107*	
C4	0.5666 (5)	0.8766 (2)	0.62260 (17)	0.0905 (11)	
H4A	0.6716	0.8988	0.6330	0.136*	
H4B	0.5689	0.8234	0.6408	0.136*	
H4C	0.4970	0.9091	0.6431	0.136*	
C5	0.5075 (3)	0.87439 (16)	0.54560 (14)	0.0609 (7)	
H5	0.5067	0.9290	0.5277	0.073*	
C6	0.3412 (4)	0.8405 (3)	0.5257 (2)	0.0934 (12)	
H6A	0.3080	0.8401	0.4763	0.140*	
H6B	0.2698	0.8729	0.5451	0.140*	
H6C	0.3404	0.7869	0.5429	0.140*	
C7	0.4706 (4)	0.38395 (19)	0.70440 (14)	0.0758 (9)	
H7A	0.3691	0.3969	0.7143	0.114*	
H7B	0.5470	0.4233	0.7248	0.114*	
H7C	0.5035	0.3323	0.7231	0.114*	
C8	0.4583 (3)	0.38282 (13)	0.62715 (12)	0.0459 (5)	
H8	0.3798	0.3424	0.6072	0.055*	
C9	0.6128 (3)	0.36272 (16)	0.60696 (15)	0.0620 (7)	
H9A	0.5979	0.3624	0.5576	0.093*	
H9B	0.6479	0.3110	0.6247	0.093*	
H9C	0.6911	0.4020	0.6257	0.093*	
C10	0.1143 (3)	0.44046 (19)	0.58522 (16)	0.0659 (7)	
H10A	0.1346	0.3909	0.6098	0.099*	
H10B	0.1027	0.4309	0.5367	0.099*	
H10C	0.0185	0.4637	0.5939	0.099*	
C11	0.2517 (3)	0.49718 (14)	0.60939 (12)	0.0461 (5)	
H11	0.2637	0.5058	0.6591	0.055*	
C12	0.2265 (3)	0.57729 (16)	0.57311 (16)	0.0597 (6)	
H12A	0.3155	0.6114	0.5903	0.090*	
H12B	0.1316	0.6015	0.5817	0.090*	
H12C	0.2166	0.5697	0.5244	0.090*	
C13	0.9325 (4)	0.76767 (18)	0.7572 (2)	0.0849 (10)	
H13A	0.8896	0.7448	0.7938	0.127*	
H13B	0.8580	0.7612	0.7142	0.127*	
H13C	0.9526	0.8235	0.7660	0.127*	
C14	1.0867 (3)	0.72570 (14)	0.75319 (14)	0.0547 (6)	
H14	1.1609	0.7320	0.7976	0.066*	
C15	1.1633 (4)	0.75867 (18)	0.69695 (19)	0.0760 (9)	
H15A	1.2604	0.7306	0.6970	0.114*	
H15B	1.1854	0.8144	0.7051	0.114*	
H15C	1.0926	0.7519	0.6530	0.114*	
C16	1.0647 (3)	0.5999 (2)	0.86078 (14)	0.0712 (8)	
H16A	1.0666	0.6552	0.8738	0.107*	
H16B	1.1714	0.5808	0.8655	0.107*	
H16C	1.0113	0.5694	0.8901	0.107*	
C17	0.9777 (2)	0.59106 (15)	0.78665 (13)	0.0476 (5)	
H17	0.8696	0.6116	0.7824	0.057*	
C18	0.9683 (3)	0.50527 (16)	0.76210 (19)	0.0720 (8)	
H18A	0.9115	0.5029	0.7150	0.108*	
H18B	0.9139	0.4738	0.7904	0.108*	
H18C	1.0735	0.4847	0.7653	0.108*	

### Atomic displacement parameters (Å^2^)

**Table:**

	*U*^11^	*U*^22^	*U*^33^	*U*^12^	*U*^13^	*U*^23^
S1	0.0348 (2)	0.0397 (3)	0.0275 (2)	−0.00589 (19)	0.00538 (17)	0.00145 (19)
S2	0.0328 (2)	0.0516 (3)	0.0309 (2)	0.0025 (2)	0.00650 (18)	−0.0062 (2)
O1	0.102 (6)	0.046 (3)	0.155 (7)	0.025 (4)	0.081 (5)	0.045 (4)
O2	0.077 (5)	0.100 (5)	0.028 (2)	−0.031 (4)	0.011 (3)	−0.018 (3)
O3	0.029 (2)	0.140 (8)	0.046 (3)	−0.017 (3)	0.0059 (19)	−0.009 (4)
O4	0.0605 (9)	0.0598 (10)	0.0357 (8)	−0.0112 (8)	0.0203 (7)	−0.0017 (7)
O1'	0.050 (3)	0.056 (3)	0.127 (5)	−0.015 (3)	−0.011 (4)	0.045 (3)
O2'	0.100 (6)	0.051 (3)	0.044 (3)	0.002 (3)	0.014 (3)	−0.011 (2)
O3'	0.057 (3)	0.096 (5)	0.045 (3)	−0.040 (3)	−0.008 (2)	0.025 (3)
O5	0.0340 (7)	0.0676 (11)	0.0536 (10)	−0.0023 (7)	0.0058 (6)	−0.0119 (8)
O6	0.0698 (11)	0.0832 (13)	0.0400 (9)	0.0056 (10)	0.0138 (8)	−0.0220 (9)
O7	0.0715 (11)	0.0512 (10)	0.0520 (10)	−0.0055 (8)	0.0265 (9)	−0.0041 (8)
O8	0.0450 (9)	0.0744 (12)	0.0647 (12)	0.0125 (8)	0.0037 (8)	0.0073 (9)
N1	0.0502 (10)	0.0411 (10)	0.0312 (9)	−0.0023 (8)	0.0068 (7)	0.0063 (7)
N2	0.0391 (9)	0.0437 (10)	0.0297 (9)	−0.0076 (7)	0.0046 (7)	0.0014 (7)
N3	0.0317 (8)	0.0428 (9)	0.0365 (9)	0.0022 (7)	0.0010 (7)	−0.0008 (7)
C1	0.099 (2)	0.0678 (19)	0.076 (2)	−0.0386 (17)	0.0171 (17)	−0.0039 (16)
C2	0.0525 (12)	0.0613 (14)	0.0389 (12)	−0.0140 (11)	0.0031 (9)	0.0054 (10)
C3	0.0594 (15)	0.0784 (19)	0.083 (2)	−0.0034 (14)	0.0311 (14)	0.0107 (16)
C4	0.120 (3)	0.101 (3)	0.0549 (18)	0.025 (2)	0.0283 (18)	−0.0156 (17)
C5	0.0791 (18)	0.0545 (14)	0.0517 (15)	0.0212 (13)	0.0196 (12)	0.0102 (11)
C6	0.0603 (17)	0.137 (3)	0.087 (2)	0.031 (2)	0.0243 (16)	0.030 (2)
C7	0.114 (2)	0.0731 (19)	0.0387 (14)	0.0098 (17)	0.0132 (14)	0.0174 (13)
C8	0.0604 (13)	0.0373 (11)	0.0375 (11)	−0.0082 (10)	0.0044 (9)	0.0023 (9)
C9	0.0673 (16)	0.0542 (14)	0.0614 (16)	0.0126 (12)	0.0063 (13)	0.0080 (12)
C10	0.0451 (13)	0.0831 (19)	0.0718 (19)	−0.0161 (13)	0.0175 (12)	−0.0057 (15)
C11	0.0446 (11)	0.0612 (14)	0.0337 (11)	−0.0034 (10)	0.0109 (9)	−0.0096 (10)
C12	0.0482 (13)	0.0610 (15)	0.0682 (17)	0.0057 (11)	0.0081 (11)	−0.0031 (13)
C13	0.088 (2)	0.0484 (15)	0.122 (3)	0.0157 (15)	0.030 (2)	−0.0075 (17)
C14	0.0540 (13)	0.0440 (12)	0.0603 (15)	−0.0026 (10)	−0.0017 (11)	−0.0095 (11)
C15	0.0704 (18)	0.0574 (17)	0.098 (2)	−0.0149 (14)	0.0111 (16)	0.0124 (16)
C16	0.0617 (15)	0.109 (2)	0.0455 (14)	0.0151 (15)	0.0178 (12)	0.0147 (15)
C17	0.0341 (10)	0.0587 (13)	0.0511 (13)	0.0062 (9)	0.0114 (9)	0.0079 (10)
C18	0.0656 (17)	0.0508 (15)	0.100 (3)	−0.0043 (13)	0.0192 (16)	0.0134 (15)

### Geometric parameters (Å, °)

**Table:**

S1—O3	1.445 (4)	C6—H6A	0.9600
S1—O3'	1.444 (4)	C6—H6B	0.9600
S1—O1	1.448 (4)	C6—H6C	0.9600
S1—O2	1.450 (5)	C7—C8	1.513 (3)
S1—O1'	1.452 (4)	C7—H7A	0.9600
S1—O2'	1.456 (4)	C7—H7B	0.9600
S1—O4	1.4848 (15)	C7—H7C	0.9600
S2—O8	1.4344 (17)	C8—C9	1.506 (4)
S2—O5	1.4494 (16)	C8—H8	0.9800
S2—O6	1.4508 (17)	C9—H9A	0.9600
S2—O7	1.5627 (18)	C9—H9B	0.9600
O7—H7	0.856 (10)	C9—H9C	0.9600
N1—C5	1.502 (3)	C10—C11	1.516 (3)
N1—C2	1.500 (3)	C10—H10A	0.9600
N1—H1n1	0.860 (10)	C10—H10B	0.9600
N1—H1n2	0.854 (10)	C10—H10C	0.9600
N2—C8	1.504 (3)	C11—C12	1.516 (4)
N2—C11	1.506 (3)	C11—H11	0.9800
N2—H2n1	0.849 (10)	C12—H12A	0.9600
N2—H2n2	0.851 (10)	C12—H12B	0.9600
N3—C17	1.501 (3)	C12—H12C	0.9600
N3—C14	1.501 (3)	C13—C14	1.519 (4)
N3—H3n1	0.856 (10)	C13—H13A	0.9600
N3—H3n2	0.857 (10)	C13—H13B	0.9600
C1—C2	1.515 (4)	C13—H13C	0.9600
C1—H1A	0.9600	C14—C15	1.513 (4)
C1—H1B	0.9600	C14—H14	0.9800
C1—H1C	0.9600	C15—H15A	0.9600
C2—C3	1.511 (4)	C15—H15B	0.9600
C2—H2	0.9800	C15—H15C	0.9600
C3—H3A	0.9600	C16—C17	1.512 (4)
C3—H3B	0.9600	C16—H16A	0.9600
C3—H3C	0.9600	C16—H16B	0.9600
C4—C5	1.506 (4)	C16—H16C	0.9600
C4—H4A	0.9600	C17—C18	1.513 (4)
C4—H4B	0.9600	C17—H17	0.9800
C4—H4C	0.9600	C18—H18A	0.9600
C5—C6	1.516 (5)	C18—H18B	0.9600
C5—H5	0.9800	C18—H18C	0.9600
			
O3—S1—O1	110.9 (4)	C8—C7—H7A	109.5
O3—S1—O2	110.3 (4)	C8—C7—H7B	109.5
O1—S1—O2	110.9 (4)	H7A—C7—H7B	109.5
O3'—S1—O1'	109.7 (4)	C8—C7—H7C	109.5
O3'—S1—O2'	110.7 (4)	H7A—C7—H7C	109.5
O1'—S1—O2'	108.3 (4)	H7B—C7—H7C	109.5
O3—S1—O4	110.8 (4)	C9—C8—N2	107.92 (19)
O3'—S1—O4	110.3 (3)	C9—C8—C7	113.1 (2)
O1—S1—O4	107.5 (3)	N2—C8—C7	110.9 (2)
O2—S1—O4	106.4 (3)	C9—C8—H8	108.3
O1'—S1—O4	110.9 (3)	N2—C8—H8	108.3
O2'—S1—O4	106.8 (3)	C7—C8—H8	108.3
O8—S2—O5	112.37 (11)	C8—C9—H9A	109.5
O8—S2—O6	114.51 (11)	C8—C9—H9B	109.5
O5—S2—O6	112.38 (10)	H9A—C9—H9B	109.5
O8—S2—O7	107.02 (11)	C8—C9—H9C	109.5
O5—S2—O7	106.37 (10)	H9A—C9—H9C	109.5
O6—S2—O7	103.28 (11)	H9B—C9—H9C	109.5
S2—O7—H7	112 (3)	C11—C10—H10A	109.5
C5—N1—C2	118.4 (2)	C11—C10—H10B	109.5
C5—N1—H1n1	107.6 (17)	H10A—C10—H10B	109.5
C2—N1—H1n1	106.2 (16)	C11—C10—H10C	109.5
C5—N1—H1n2	107.9 (18)	H10A—C10—H10C	109.5
C2—N1—H1n2	108.1 (18)	H10B—C10—H10C	109.5
H1n1—N1—H1n2	108 (2)	N2—C11—C10	110.6 (2)
C8—N2—C11	118.61 (18)	N2—C11—C12	107.53 (19)
C8—N2—H2n1	105.4 (18)	C10—C11—C12	112.3 (2)
C11—N2—H2n1	106.8 (18)	N2—C11—H11	108.8
C8—N2—H2n2	106.2 (17)	C10—C11—H11	108.8
C11—N2—H2n2	110.3 (17)	C12—C11—H11	108.8
H2n1—N2—H2n2	109 (3)	C11—C12—H12A	109.5
C17—N3—C14	118.56 (19)	C11—C12—H12B	109.5
C17—N3—H3n1	108.2 (17)	H12A—C12—H12B	109.5
C14—N3—H3n1	106.2 (16)	C11—C12—H12C	109.5
C17—N3—H3n2	108.2 (17)	H12A—C12—H12C	109.5
C14—N3—H3n2	108.2 (16)	H12B—C12—H12C	109.5
H3n1—N3—H3n2	107 (2)	C14—C13—H13A	109.5
C2—C1—H1A	109.5	C14—C13—H13B	109.5
C2—C1—H1B	109.5	H13A—C13—H13B	109.5
H1A—C1—H1B	109.5	C14—C13—H13C	109.5
C2—C1—H1C	109.5	H13A—C13—H13C	109.5
H1A—C1—H1C	109.5	H13B—C13—H13C	109.5
H1B—C1—H1C	109.5	N3—C14—C15	107.5 (2)
N1—C2—C3	107.7 (2)	N3—C14—C13	110.6 (2)
N1—C2—C1	111.4 (2)	C15—C14—C13	112.9 (3)
C3—C2—C1	112.3 (2)	N3—C14—H14	108.6
N1—C2—H2	108.5	C15—C14—H14	108.6
C3—C2—H2	108.5	C13—C14—H14	108.6
C1—C2—H2	108.5	C14—C15—H15A	109.5
C2—C3—H3A	109.5	C14—C15—H15B	109.5
C2—C3—H3B	109.5	H15A—C15—H15B	109.5
H3A—C3—H3B	109.5	C14—C15—H15C	109.5
C2—C3—H3C	109.5	H15A—C15—H15C	109.5
H3A—C3—H3C	109.5	H15B—C15—H15C	109.5
H3B—C3—H3C	109.5	C17—C16—H16A	109.5
C5—C4—H4A	109.5	C17—C16—H16B	109.5
C5—C4—H4B	109.5	H16A—C16—H16B	109.5
H4A—C4—H4B	109.5	C17—C16—H16C	109.5
C5—C4—H4C	109.5	H16A—C16—H16C	109.5
H4A—C4—H4C	109.5	H16B—C16—H16C	109.5
H4B—C4—H4C	109.5	N3—C17—C16	110.7 (2)
N1—C5—C4	111.5 (2)	N3—C17—C18	107.4 (2)
N1—C5—C6	107.2 (2)	C16—C17—C18	112.8 (2)
C4—C5—C6	112.1 (3)	N3—C17—H17	108.6
N1—C5—H5	108.6	C16—C17—H17	108.6
C4—C5—H5	108.6	C18—C17—H17	108.6
C6—C5—H5	108.6	C17—C18—H18A	109.5
C5—C6—H6A	109.5	C17—C18—H18B	109.5
C5—C6—H6B	109.5	H18A—C18—H18B	109.5
H6A—C6—H6B	109.5	C17—C18—H18C	109.5
C5—C6—H6C	109.5	H18A—C18—H18C	109.5
H6A—C6—H6C	109.5	H18B—C18—H18C	109.5
H6B—C6—H6C	109.5		
			
C5—N1—C2—C3	−180.0 (2)	C8—N2—C11—C10	−58.1 (3)
C5—N1—C2—C1	−56.5 (3)	C8—N2—C11—C12	179.04 (18)
C2—N1—C5—C4	−53.9 (3)	C17—N3—C14—C15	−177.8 (2)
C2—N1—C5—C6	−176.9 (2)	C17—N3—C14—C13	−54.1 (3)
C11—N2—C8—C9	−178.90 (19)	C14—N3—C17—C16	−55.2 (3)
C11—N2—C8—C7	−54.6 (3)	C14—N3—C17—C18	−178.7 (2)

### Hydrogen-bond geometry (Å, °)

**Table:**

*D*—H···*A*	*D*—H	H···*A*	*D*···*A*	*D*—H···*A*
O7—H7···O4	0.86 (1)	1.76 (1)	2.609 (2)	174 (4)
N1—H1N1···O1	0.86 (1)	1.76 (1)	2.585 (5)	159 (3)
N1—H1N2···O6^i^	0.85 (1)	2.02 (1)	2.874 (2)	176 (3)
N2—H2N2···O4	0.85 (1)	2.10 (1)	2.929 (2)	166 (2)
N2—H2N1···O2^ii^	0.85 (1)	1.85 (1)	2.695 (6)	179 (3)
N2—H2N1···O2'^ii^	0.85 (1)	2.02 (2)	2.855 (8)	166 (3)
N3—H3N1···O3	0.86 (1)	1.89 (1)	2.738 (8)	174 (2)
N3—H3N1···O3'	0.86 (1)	1.88 (1)	2.711 (7)	162 (2)
N3—H3N2···O5^iii^	0.86 (1)	2.00 (1)	2.819 (2)	159 (2)

Symmetry codes: (i) *x*, −*y*+3/2, *z*−1/2; (ii) −*x*+1, −*y*+1, −*z*+1; (iii) *x*+1, *y*, *z*.
